# Diffuse scaly erythematous plaques in patient taking poziotinib

**DOI:** 10.1016/j.jdcr.2021.09.042

**Published:** 2021-11-04

**Authors:** Reid Oldenburg, Maha Albukhari, Brianne H. Daniels, Amanda F. Marsch

**Affiliations:** a Department of Dermatology, University of California San Diego, La Jolla, San Diego, California; b College of Medicine, Princess Nourah Bint Abdulrahman University, Riyadh, Saudi Arabia

**Keywords:** acantholysis, EGFR/HER inhibitor, papulopustular eruption, poziotinib, tyrosine kinase inhibitor, EGFR, epidermal growth factor receptor

A 77-year-old man with recurrent lung adenocarcinoma presented with a progressive rash 10 weeks after starting poziotinib 8 mg twice daily. The rash started on his face and progressed to his chest, back, hands and buttocks despite the use of doxycycline 100 mg twice daily and topical hydrocortisone 2.5% ointment for 4 weeks. Physical examination revealed diffuse excoriated erythematous acneiform papules and plaques on his trunk, arms, and buttocks. Diffuse honey-colored crusted erythematous plaques and bilateral conjunctival erythema were found on his face (Fig 1). Biopsy of the right aspect of upper back demonstrated focal neutrophilic folliculitis with epidermal atrophy with focal acantholysis (Fig 2).

**Fig 1:**
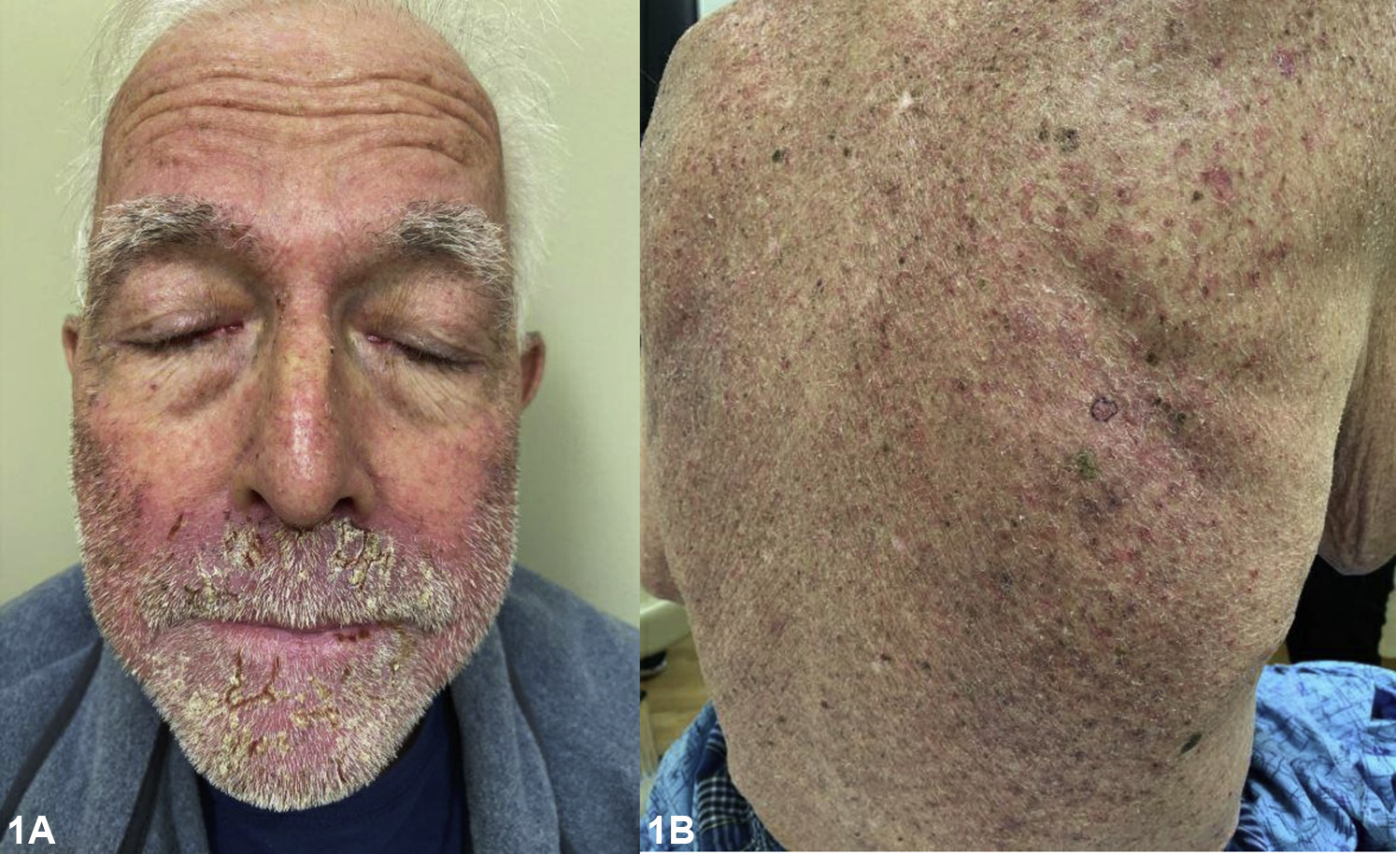

**Fig 2:**
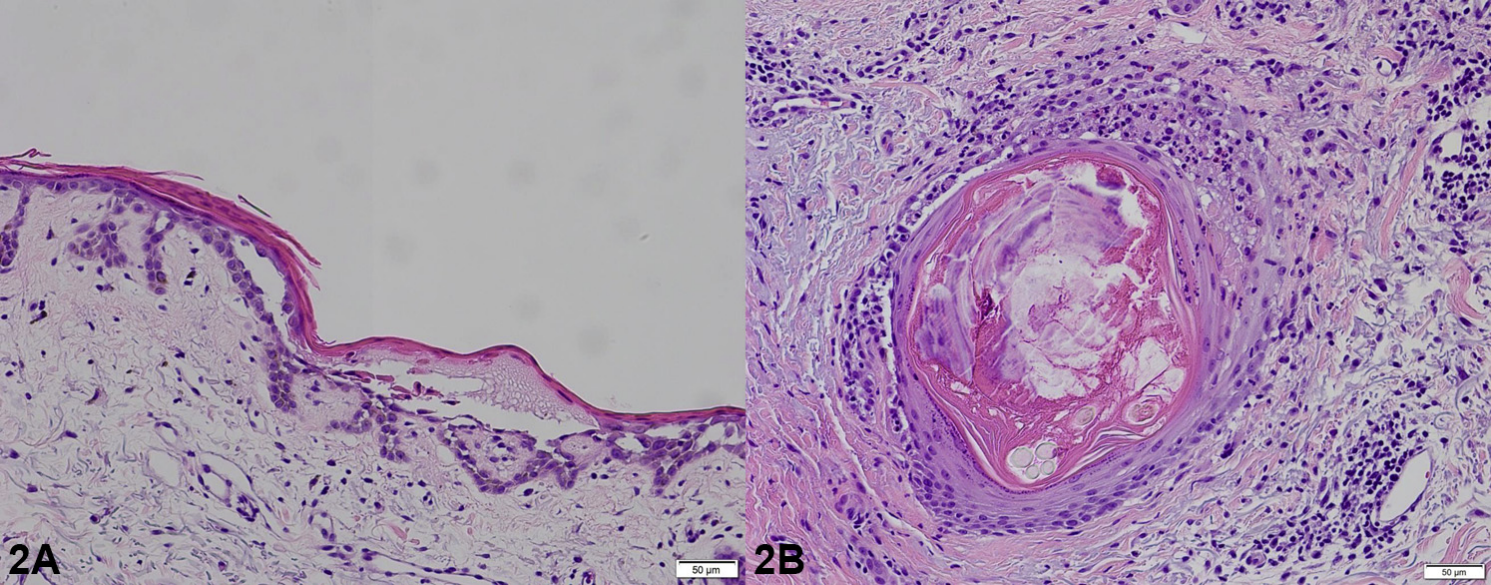

**Question 1: What is the most likely diagnosis?**

A. Seborrheic dermatitis
B. Pityrosporum folliculitis
C. Drug-induced papulopustular eruption with bacterial superinfection
D. Drug-induced hypersensitivity syndrome
E. Eosinophilic pustular folliculitis

**Answers:**

A. Seborrheic dermatitis – Incorrect. Cutaneous findings of seborrheic dermatitis are characterized by a yellow greasy scale in a seborrheic distribution.
B. Pityrosporum folliculitis – Incorrect. Pityrosporum folliculitis presents with pruritic, fine monomorphic papules and pustules on the face and back and may be confused with acne vulgaris.
C. Drug-induced papulopustular eruption with bacterial superinfection – Correct. Papulopustular drug eruptions, also known as acneiform drug eruptions, are common adverse reactions triggered by chemotherapeutic agents that target epidermal growth factor receptor (EGFR) family signaling pathways.^1^ Papulopustular drug eruptions usually present as diffuse excoriated erythematous papules and pustules that can coalesce into scaly plaques. Pustules are typically sterile, but significant superinfection can occur.^2^
D. Drug-induced hypersensitivity syndrome – Incorrect. This condition typically presents as an extensive morbilliform eruption and is often accompanied by lymphadenopathy, fever, pharyngitis, and laboratory-based evidence of end-organ damage.
E. Eosinophilic pustular folliculitis – Incorrect. This condition classically presents as an eruption of papulopustules over erythematous annular plaques with associated pruritus. Other diagnoses with similar morphology, such as acne vulgaris, bacterial folliculitis, dermatophyte infection, mycosis fungoides, seborrheic dermatitis, and rosacea can be excluded with a skin biopsy submitted to potassium hydroxide examination.

**Question 2: How does poziotinib differ from panitumumab and cetuximab?**

A. Poziotinib inhibits EGFR selectively
B. Poziotinib is a monoclonal antibody
C. Poziotinib inhibits dihydrofolate reductase
D. Poziotinib is a small-molecule inhibitor of PI3K
E. Poziotinib targets multiple members of the EGFR family

**Answers:**

A. Poziotinib inhibits EGFR selectively – Incorrect. Poziotinib, panitumumab, and cetuximab all target EGFR.^3^
B. Poziotinib is a monoclonal antibody – Incorrect. Poziotinib is an irreversible tyrosine kinase inhibitor.^4^ Panitumumab and cetuximab are monoclonal antibodies that target EGFR and used to treat tumors that do not harbor downstream RAS gene mutations.^3^
C. Poziotinib inhibits dihydrofolate reductase – Incorrect. Poziotinib, panitumumab, and cetuximab do not directly target pathways involved in purine synthesis.
D. Poziotinib is a small-molecule inhibitor of PI3K – Incorrect. Poziotinib, panitumumab, and cetuximab do not target PI3K.
E. Poziotinib targets multiple members of the EGFR family – Correct. Panitumumab and cetuximab are monoclonal antibodies that target EGFR.^3^ Poziotinib is an irreversible tyrosine kinase inhibitor that targets EGFR, HER2, and HER4.^4^ In a study of 106 patients treated with poziotinib, 32.1% of patients experienced acneiform eruptions and 63.2% experienced pruritus.^4^ In a meta-analysis of 10,379 patients, 85.3% of patients taking cetuximab and 77% of patients taking panitumumab experienced acneiform eruptions; 17.4% of patients taking cetuximab and 32% of patients taking panitumumab experienced pruritus.^3^

**Question 3: What is the most common organism found in this condition?**

A. *Malassezia furfur*
B. Group A *Streptococcus*
C. *Candida albicans*
D. *Staphylococcus aureus*
E. *Pseudomonas aeruginosa*

**Answers:**

A. *Malassezia furfur* – Incorrect. There are no reported cases of *M furfur* superinfection occurring in drug-induced papulopustular eruptions.
B. Group A *Streptococcus* – Incorrect. While Group A *Streptococcus* superinfection is a possibility, there are no reported cases.
C. *Candida albicans* – Incorrect. In a retrospective chart review of 88 patients, only 1 patient had *C albicans* superinfection of the skin. However, *C albicans* superinfection can occur in onychomycosis or paronychia seen in patients taking EGFR inhibitors.^2^
D. *Staphylococcus aureus* – Correct. This is the most reported organism to cause superinfection in EGFR inhibitor-induced skin reactions.^2^ Methicillin and tetracycline resistance has been reported, so it is critical to obtain a culture and sensitivity to determine appropriate antibiotic therapy.^5^
E. *Pseudomonas aeruginosa* – Incorrect. There are reported cases of superinfection with *P aeruginosa* in patients with EGFR inhibitor-induced skin reactions, but it is not the most common causative organism. However, this case highlights the importance of obtaining a bacterial culture and sensitivity, since this was the culprit pathogen in this patient.

## Conflicts of interest

None disclosed.
